# Induction of viral and tumour specific CTL responses using antibody targeted HLA class I peptide complexes

**DOI:** 10.1038/sj.bjc.6600223

**Published:** 2002-04-22

**Authors:** P Savage, P Cowburn, A Clayton, S Man, A McMichael, N Lemoine, A Epenetos, G Ogg

**Affiliations:** Alexis Biotechnology, 81 Harley Street, London W1N 1DE, UK; Cancer Research Wales, Velindre Hospital, Whitchurch, Cardiff CF14 2TL, UK; Department of Medicine, University of Wales College of Medicine, Cardiff CF14 2TL, UK; MRC, Human Immunology Unit, IMM, Oxford OX3 9DS, UK; ICRF Molecular Oncology Unit, Hammersmith Hospital, London W12 0NN, UK; Department of Medical Oncology, St Bartholomews Hospital, London EC1A 7BE, UK

**Keywords:** cancer, immunotherapy, HLA class I, vaccines, B cells, CD20

## Abstract

The production of cytotoxic T cells with specificity for cancer cells is a rapidly evolving branch of cancer therapeutics. A variety of approaches aim to amplify anti-tumour cytotoxic T cell responses using purified peptides, tumour cell lysates or recombinant HLA/peptide complexes in differing antigen presenting systems. Using a two-step biotin-streptavidin antibody targeting system, recombinant HLA-class I/peptide complexes were attached to the surface of B cells via the anti-CD20 B9E9-scFvSA antibody-streptavidin fusion protein. Flow cytometry with a conformation dependant monoclonal antibody to HLA class I indicated that targeted HLA-class I/peptide complexes remain on the surface of B cells in culture for periods in excess of 72 h. PBMCs were stimulated *in vitro* for 8–14 days using the autologous B cells as antigen presenting cells. Following a single cycle of stimulation specific cytotoxic T cell responses to targeted HLA-A2 complexes containing the M1, BMLF1 and Melan A peptides could be demonstrated by tetramer staining and Cr release assays. With the HLA-A2/BMLF1 complex up to 2.99% of CD8+ve cells were tetramer positive producing 20% lysis (E : T 10 : 1) of CIR-A2 target cells in an *in vitro* cytotoxicity assay compared to baseline levels of 0.09% tetramer +ve and 2% lysis in the unstimulated population. PBMCs from a healthy donor treated with two cycles of stimulations with targeted HLA-A2/Melan A complexes, demonstrated expansion of the melanA tetramer +ve population from 0.03% to 1.4% producing 15% lysis of Melan A pulsed target cells. With further consideration to the key variables of HLA/peptide complex density, the ratio of stimulator to effector cells and optimum cytokine support, this system should offer an easy and effective method for the *in vitro* amplification of specific cytotoxic T cell responses and warrants development for the *in vivo* induction of cytotoxic T cell responses in cancer therapy.

*British Journal of Cancer* (2002) **86**, 1336–1342. DOI: 10.1038/sj/bjc/6600223 www.bjcancer.com

© 2002 Cancer Research UK

## 

A central aim of cancer immunotherapy is the induction of effective cytotoxic T cell (CTL) activity that recognises HLA class I/peptide complexes that are either specific to or over-represented on tumour cells (Rosenberg, 1996). There is increasing evidence that low levels of CTLs specific for ‘tumour’ peptides are present in a number of malignancies (Pittet *et al*, 1999), however the magnitude of these pre-existing responses frequently appears to be insufficient for effective *in vivo* activity.

The interaction between the HLA class I/peptide complex and the T cells antigen receptor is the final pathway in the expansion of CD8 +ve CTLs. A range of approaches aim to reach this interaction, starting with either defined tumour associated peptide or more complex cellular based preparations. These methods include vaccination with peptides (Rosenberg *et al*, 1998), naked DNA (Mincheff *et al*, 2000) or irradiated tumour cells (Chan and Morton, 1998), these systems rely on processing and presentation by native antigen presenting cells (APCs). Alternatively *ex vivo* expanded dendritic cells can be used either with peptide pulsing (Hsu *et al*, 1996; Brossart *et al*, 2000), loading with tumour lysate (Nestle *et al*, 1999) or transfected with genes encoding tumour proteins (Wang *et al*, 2000a). Recombinant HLA-class I/peptide complexes either immobilised on beads (Lone *et al*, 1998; Tham *et al*, 2001), incorporated into antibody based fusion proteins (Cullen *et al*, 1999), or as recombinant MHC tetramers (Wang *et al*, 2000b) have also produced effective CTL responses both *in vitro* and in pre-clinical models. Dendritic cells are the most effective APCs but are present in low numbers *in vivo* and are difficult to culture, in contrast B cells are present in large numbers, are simple to manipulate *in vitro* and have been demonstrated to act effectively as APCs inducing specific CTL responses *in vivo* (Gajewski *et al*, 2001).

Previously it has been demonstrated that HLA class I/viral peptide complexes targeted to B cells via an antibody delivery system can serve as effective targets for the lytic action of anti-viral CTLs (Ogg *et al*, 2000; Savage *et al*, 2002). In this current study we have used a similar system to investigate if the two-step antibody delivery system (see Figure 1

**Figure 1:**
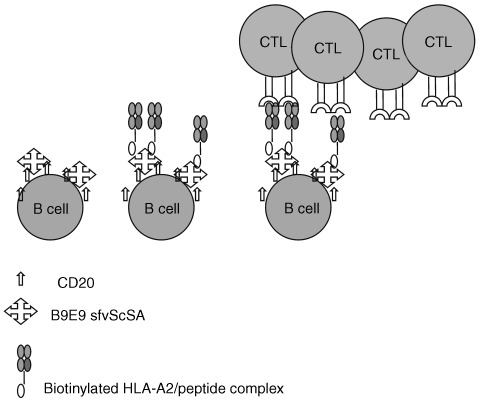
Schematic representation of the two-step targeting system delivering HLA-class I peptide complexes to the surface of B cells. Step 1 is the delivery of the anti-CD20 B9E9 sfvScSA fusion protein. Step 2 the delivery of recombinant biotinylated HLA class I peptide. These steps are followed by the selective proliferation of peptide specific CTLs.

) is able to produce the specific expansion of CTLs of chosen specificities from unselected populations of PBMCs.

## MATERIALS AND METHODS

### Antibodies

The B9E9 scFvSA fusion protein contains the single-chain variable region of the murine IgG2a anti-CD20 murine antibody B9E9 fused to the genomic streptavidin of *Streptomyces avidinii*. The protein is secreted into the periplasm of genetically engineered *E. coli* as monomeric subunits (43 400 Daltons) that spontaneously fold into soluble tetramers with a molecular weight of 173 600 Daltons. The four antigen-binding and biotin-binding sites of the fusion protein retain the functional capabilities of the parent molecules (Schultz *et al*, 2000). The FITC conjugated monoclonal antibodies used in flow cytometry were anti-MHC class I (W6/32) (Cymbus Biotechnology, Harrow, UK), anti-CD19, anti-CD80 and anti-CD86 (Dako, Ely, UK).

### Cells

The CIR-A2 (Storkus *et al*, 1989) and Daudi (Klein *et al*, 1968) cell lines were grown in RPMI + 10% FCS supplemented with Penicillin and Streptomycin in a 37°C incubator with 5% CO_2_.

PBMCs were isolated from healthy volunteers and melanoma patients previously documented to be HLA-A2 +ve. Approximately 30 mls of venous blood was obtained by venepuncture and unfractionated PBMCs were obtained by differential centrifugation using Histopaque (Sigma, Poole, UK).

### HLA-A2/peptide complex monomers and tetramers

Recombinant HLA-A2 class I molecules were obtained from ProImmune Ltd (Oxford Science Park, Oxford, UK). In brief, recombinant HLA-A2 heavy chain and beta-2 microglobulin were produced in *E. coli*. The functional HLA class I/peptide complex were produced by refolding around the peptide of choice and then biotinylation via the Bir A site on the HLA heavy chain (Garboczi *et al*, 1992; Altman *et al*, 1996). The peptides used in these experiments were Influenza virus M1 peptide GILGFVFTL (Gotch *et al*, 1987), Epstein-Barr virus (EBV) BMLF1 peptide GLCTLVAML (Steven *et al*, 1997) and the modified melanoma associated Melan A peptide ELAGIGILTV (Valmori *et al*, 1998). The PE conjugated fluorescent HLA-A2/peptide tetramers of the same specificities used for flow cytometric analysis were also obtained from ProImmune.

### Targeting of B9E9 scFvSA and HLA-A2/peptide complexes to HLA class I -ve B cells

HLA class I -ve Daudi cells were used to investigate the binding of the HLA-A2/class I peptide complexes via the B9E9 scFvSA. Cells were washed in PBS and incubated with B9E9 scFvSA diluted in PBS at 10 ug ml^−1^ for 1 h at RT. After washing the cells were incubated with either biotinylated HLA-A2/M1 peptide complexes at 0.5 μg ml^−1^ or PBS alone for 30 min at RT. After further washing the two groups of cells were resuspended in RPMI + 10% FCS and grown at 37°C in a 5% CO_2_ atmosphere. At various time points parallel samples of cells were removed, washed and incubated for 30 min at RT with FITC conjugated W6/32, after washing the cells were analysed by flow cytometry.

### The effects of B9E9 scFvSA binding on the expression of co-stimulatory molecules in PBMC B cells

PBMCs prepared by differential centrifugation were incubated with B9E9 scFvSA (10 μg ml^−1^), IL-7 (10 ng ml^−1^), B9E9 scFvSA and IL-7 or PBS alone for 1 h at RT. After washing the cells were placed into tissue culture media and returned to culture at 37°C. Samples were removed and double stained with PE conjugated anti-CD19 and either FITC conjugated anti-CD80 or anti-CD86 and analysed on a Becton Dickinson FACScan using FACScomp software.

### *In vitro* immunisation protocol

PBMCs were incubated with the B9E9 scFvSA (10 μg ml^−1^) diluted in PBS for 1 h at RT. After washing cells were incubated with the biotinylated HLA class I/peptide complex (0.5 μg ml^−1^ in PBS) for 30 min at RT. Various controls, omitting the B9E9 scFvSA or the HLA class I/peptide complex were also performed. After washing, cells were placed into 24-well plates at 3×10^6^ cells per well and cultured in RPMI with 10% human AB serum. IL-7 (R and D Systems, Minneapolis, MN, USA) was added on day 1 at 10 ng ml^−1^ and IL-2 (Chiron, Harefield, UK) was added at 10 U ml^−1^ on day 4 and every further 3 days following the method described by Lalvani *et al* (1997). In the experiments with a second stimulation cycle further PBMCs were obtained and treated as above. These new cells were then mixed with the existing culture at a 1 : 2 ratio and the culture continued for a further 8 days.

### Flow cytometry and tetramer analysis

To stain CD8 +ve cells from the PBMC culture approximately 1×10^6^ cells were washed in PBS, resuspended and incubated with tetramer solution for 30 min at 37°C followed by FITC conjugated anti-CD8 for 20 min at 4°C. After incubation the cells were washed, resuspended in PBS and analysed by dual colour flow cytometry. The results of flow cytometry analysis of dual stained PBMCs are shown with anti-CD8 (Y axis) and HLA-A2/M1 tetramers (X axis). Percentage figures relate to the number of tetramer positive CD8 +ve cells from the total CD8 +ve population.

### Chromium release assay

Daudi or CIR-A2 cells were labelled with 2 uCi/uL of ^51^Cr (Amersham Pharmacia, UK) for 1 h at 37°C then washed. Daudi cells were sequentially coated with B9E9 scFvSA and HLA-A2/M1 complexes following the method above whilst CIR-A2 cells were pulsed with the peptide of choice at a concentration of 10 uM for 1 h at 37°C. The target cells were plated at 3000 cells per well in U bottomed 96-well plates. PBMCs, media or 5% Triton X-100 were added to a final volume of 200 μl. Plates were incubated for 4 h at 37°C in a 5% CO_2_ atmosphere and 50 μl of supernatant was collected and added to 150 μl of scintillant. The specific lysis was calculated as:


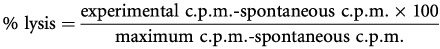


The spontaneous release was measured from the cells incubated in media alone, the maximum release was measured from the cells incubated in 5% Triton.

## RESULTS

### Sequential analysis of the binding of biotinylated HLA-A2/M1 complexes to Daudi B-cell lymphoma cells via B9E9 scFvSA

**Figure 2:**
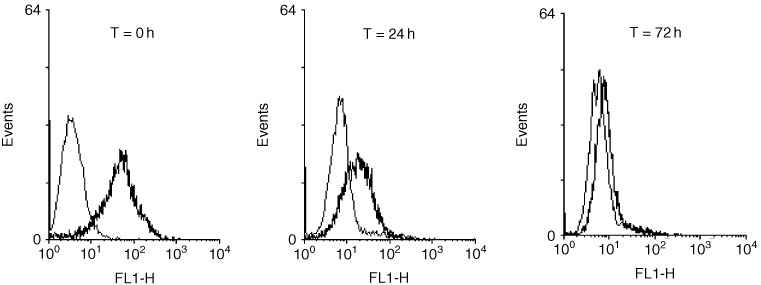
Time course analysis of HLA-A2/M1 complexes immobilised on the surface of HLA-class 1 −ve Daudi cells via B9E9 sfvScSA. Complexes are detected via the binding of W6/32 which binds conformationally correct HLA-class I. Daudi cells targeted with B9E9 sfvScSA alone are shown in grey, in black are Daudi cells targeted with B9E9 sfvScSA and HLA-A2/M1.

The time course of the retention of the targeted HLA-A2/M1 complexes retention the HLA class I -ve Daudi cells is demonstrated in the sequential flow cytometry analyses in Figure 2

. An increased fluorescence signal is demonstrated in the targeted cells which decreases with time. However a positive signal is still present at 72 h and it is probable that HLA class I/peptide complexes persist at functional levels beyond this time.

### Effects of B9E9 scFvSA binding on the expression of co-stimulatory molecules in PBMC B cells

Figure 3

**Figure 3:**
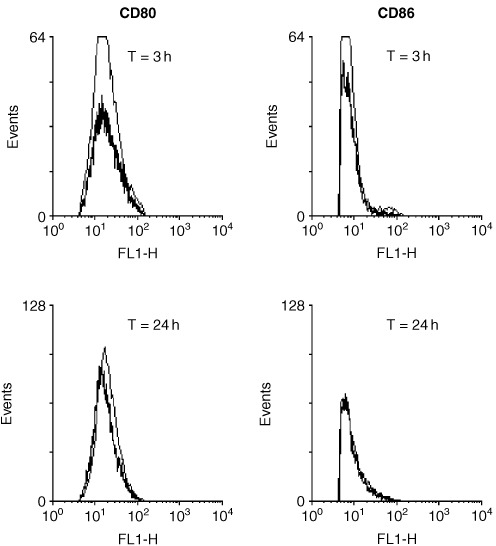
Sequential flow cytometry analysis of expression of CD80 and CD86 on CD19 +ve B cells within the PBMC population. The unstimulated controls are shown on the narrow trace, the experimental results from PBMCs targeted with the B9E9 sfvScSA are shown in bold.

demonstrates that the addition of B9E9 scFvSA has no detectable effect on the expression of CD80 or CD86 on the B cells within the PBMC population. The results show the flow cytometry results for CD19 +ve cells at 3 h and 24 h. PBMCs treated with IL-7 alone or the combination of B9E9 scFvSA and IL-7 also demonstrated no change in the levels of expression of CD80 and CD86 (data not shown).

### Induction of CTL activity with targeted HLA class I/peptide complexes

**Figure 4:**
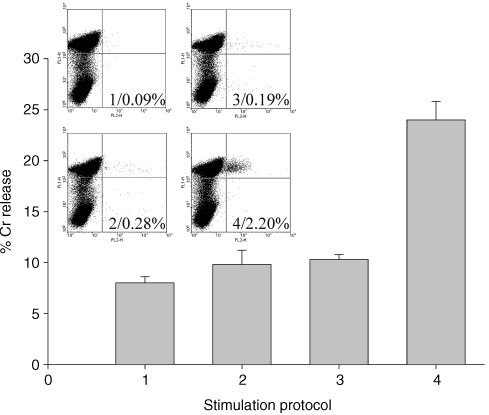
FACs and Cr release assay results from PBMCs stimulated with targeted HLA-A2/M1 complexes. Tetramer results demonstrate staining with anti-CD8 and HLA-A2/M1 tetramer. The Cr release assay demonstrates activity against Daudi cells bearing HLA-A2/M1 complexes. Stimulation protocols:1/ PBMCs alone, 2/ PBMCs + B9E9 sfvScSA, 3/ PBMCs + free HLA-A2/BMLF1, 4/ PBMCs + B9E9 sfvScSA + HLA-A2/BMLF1.

The ability of the antibody targeted complexes to stimulate CTL expansion was initially examined with the HLA-A2/M1 combination. In Figure 4

the tetramer analysis of the CD8+ve/HLA-A2/M1 positive cells within the unstimulated PBMCs (1), PBMCs targeted with the B9E9 scFvSA (2), and PBMCs exposed to free soluble HLA-A2/M1 complexes at 0.1 ng ml^−1^ (3) demonstrate values of 0.06% to 0.22%. In contrast the PBMCs targeted with the B9E9 scFvSA and HLA-A2/M1 complexes (4) demonstrated 2.33% tetramer positive CD8+ve cells. Using the unfractionated PBMCs at E : T 10 : 1, a 4 h Cr release assay, using HLA-A2/M1 coated Daudi cells as target cells, demonstrated a maximum of 10% lysis from the three control experiments but 24% from the PBMCs stimulated with HLA-A2/M1 complexes attached via the B9E9 scFvSA fusion protein.

### Induced CTL responses are specific for the targeted complex

To confirm the specificity of CTL expansion, PBMCs were targeted with either B9E9 scFvSA alone (A) or B9E9 scFvSA and HLA-A2/BMLF1 complexes (B).

In Figure 5

**Figure 5:**
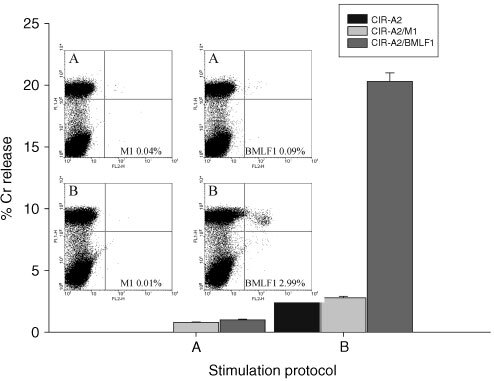
FACs and Cr release assay results from PBMCs stimulated with either (**A**) B9E9 sfvScSA alone or (**B**) B9E9 sfvScSA and targeted HLA-A2/BMLF1 complexes. Tetramer staining results are shown for both HLA-A2/M1 and HLA-A2/BMLF1. The Cr release assay demonstrates PBMC at E : T 10 : 1 against native/M1/BMLF1 peptide pulsed CIR-A2 target cells.

the tetramer analysis of the PBMCs targeted with B9E9 scFvSA alone demonstrates background staining of 0.04% with HLA-A2/M1 and 0.09% with HLA-A2/BMLF1. In the Cr release assay against CIR-A2 cells either native or pulsed with M1 or BMLF1 peptide the PBMCs showed no significant activity. In contrast PBMCs targeted with the HLA-A2/BMLF1 complexes demonstrate 2.99% staining with the HLA-A2/BMLF1 tetramer but with only a background staining level of 0.01% with the HLA-A2/M1 tetramer. These cells produced 20% lysis of the BMLF1 pulsed CIR-A2 target cells without any significant action on native or M1 pulsed cells.

### CTL responses to a single cycle of stimulation with HLA-A2/peptide complexes in healthy donors and melanoma patients

The numerical values of the tetramer results from PBMCs from a series of healthy donors and melanoma patients are demonstrated in Table 1

**Table 1:**
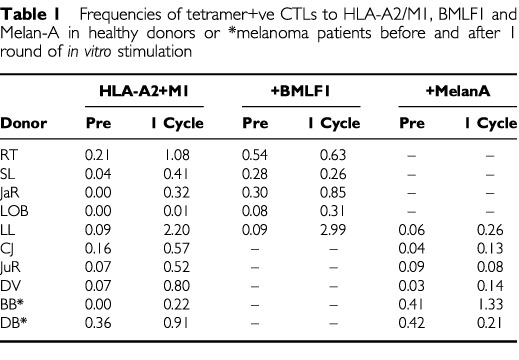
Frequencies of tetramer+ve CTLs to HLA-A2/M1, BMLF1 and Melan-A in healthy donors or *melanoma patients before and after 1 round of *in vitro* stimulation

. In response to stimulation with targeted HLA-A2/M1 complexes a greater than five-fold increase in the number of tetramer +ve cells are seen in six of the eight volunteers and one of two melanoma patients. From the HLA-A2/BMLF1 stimulated cells one of the five volunteers showed a greater than five-fold increase with two others showing apparent increases. In response to targeted HLA-A2/Melan A complexes greater than three-fold increases in tetramer positive cells were seen in three of four volunteers and in one of the melanoma patients.

### CTL responses to HLA-A2/M1 and Melan A can be enhanced by a repeated stimulation

Figure 6

**Figure 6:**
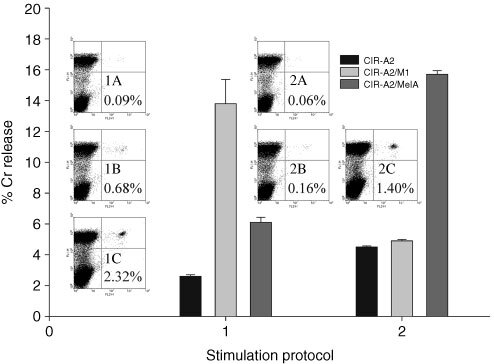
FACs and Cr release assay results from PBMCs stimulated twice with either (1) targeted HLA-A2/M1 complexes or (2) targeted HLA-A2/Melan A complexes. Tetramer results are (**A**) unstimulated, (**B**) targeted once and (**C**) targeted twice. The Cr release assay with twice targeted PBMCs shows the activity against native and peptide pulsed CIR-A2 cells at E : T 20 : 1.

demonstrates the CTL responses produced by two rounds of *in vitro* stimulation using the same HLA-A2/peptide complex. The 4 h Cr release assay (E : T 20 : 1) demonstrates that PBMCs stimulated with targeted HLA-A2/M1 complexes on both day 1 and day 8 produce 14% lysis of the CIR-A2 M1 pulsed cells compared with 3% lysis of native and 6% lysis of CIR-A2 melanA pulsed cells. The increase in HLA-A2/M1 specific CTLs is shown in the tetramer series with 0.09% from unstimulated cells (1A), 0.68% after one cycle (1B) and 2.32% after two cycles (1C). In this donor similar results were seen with responses to melan A with a 15% lysis of CIR-A2 cells pulsed with the Melan A peptide and increases in tetramer staining from a background of 0.06%, 0.16% after one cycle and 1.40% after two cycles. In this experiment cells subject to one cycle of stimulation did not produce detectable activity in the Cr release assay (data not shown).

## DISCUSSION

The induction of an effective immune response against malignant cells has been an aim of cancer research for over a century. With the increasing understanding of how the immune system can differentiate between normal and malignant cells a number of cancer vaccine approaches have been examined. To date many of these have centred on the use of undefined antigens via tumour cell lysates or irradiated cells. However with the identification of a number of potential tumour peptide epitopes (Boon and van der Bruggen, 1996; Vonderheide *et al*, 1999) and the ease of manufacture of recombinant HLA class I peptide complexes (Garboczi *et al*, 1992) it is now feasible to consider highly specific approaches to cancer vaccine strategies.

Purified immobilised HLA class I/peptide complexes have been shown to interact and stimulate CTLs when attached to tissue culture plates (Kane *et al*, 1989), chemically attached to cells (Anjuere *et al*, 1995; Walter *et al*, 1997) or when coated onto beads (Motta *et al*, 1998). More recently antibody targeted HLA class I/peptide complexes have been demonstrated to successfully interact with CTLs to permit lysis of targeted cells *in vitro* (Ogg *et al*, 2000; Robert *et al*, 2000).

The ability of B cells targeted with HLA class I/peptide complexes to induce CTL responses is clearly shown in Figures 4, 5 and 6. *In vitro* CTL responses demonstrated by tetramer and Cr release assays were obtained when the HLA class I/peptide complexes were targeted to PBMCs pre-treated with the B9E9 scFvSA fusion protein. In contrast free HLA class I/peptide complexes produced no apparent responses indicating the requirement for either multimerisation or immobilisation of HLA class I/peptide complexes for effective CTL stimulation as previously described (Abastado *et al*, 1995; Motta *et al*, 1998). The specificity of the CTL expansion is confirmed by the results shown in Figure 5 where increases in tetramer staining and lysis of peptide pulsed target cells was only seen in response to stimulation with that specific HLA/peptide complex. The ability of this system to further increase responses by repeated stimulation is shown in the tetramer stain results of Figure 6. Here the frequencies of CTLs reactive with HLA-A2/M1 increase from 0.09% to 2.32% after a second cycle. In this donor the MelanA results of 0.06% and 1.4% show as similar pattern, with positive Cr release assays after two cycles.

The efficiency of CTL induction has previously been shown to be related to the stability and length of expression of the HLA class-I complex on the surface of antigen presenting cells (Van der Burg *et al*, 1996; Valmori *et al*, 1998; Micheletti *et al*, 1999). In this antibody targeting system we have aimed to optimise the time course for expression of the HLA class I complexes, by using complexes with documented long half lives and a high affinity binding system to a non-internalising B cell marker. The ability of these complexes to persist in a conformationally correct form for at least 72 h on the surface of the B cells is demonstrated in Figure 2. It is probable that functionally active levels of complexes remain on the surface of the B cells for a longer period as we have previously shown that CTLs can interact efficiently with B cells with levels of targeted HLA below that detectable by flow cytometry (Savage *et al*, 2002).

The data in Figure 3 demonstrates that binding of the B9E9 scFvSA to the B cells within the PBMCs either alone or in conjunction with IL-7 had no effect on the expression of B80 and B86. Whilst the enhanced expression of co-stimulatory molecules generally increases CTL responses, it has previously been demonstrated that effective CTL can be produced without accessory molecule expression, particularly when high epitope densities are used (Wang *et al*, 2000b).

At present there has been only limited optimisation of this *in vitro* stimulation protocol and it is apparent from Table 1 that there is considerable variation in the level of CTL responses produced from different individuals. However the ability to produce CTL responses to viral peptides in the majority of donors from a single round of *in vitro* immunisation compares favourably with *in vitro* results from both peptide pulsing (Lalvani *et al*, 1997) and dendritic cells (Larsson *et al*, 2000). Further work is in progress to optimise the system, however it was observed in the experimental cultures using targeted HLA class I/peptide complexes there was significant inhibition of proliferation compared to those targeted with the B9E9 fusion protein alone (data not shown). It is possible that the effect of the supra-physiological stimulation prevents expansion of the specific CTL population either via a direct apoptotic action or the result of high levels of cytokine production within the closed system. Studies in mice have shown that increased antigenic density can result in higher CTL activity but produces a significant reduction at very high densities (Wherry *et al*, 1999). Similarly in human systems the presence of supra-optimal levels of HLA class I complexes can lead to apoptosis rather than expansion of stimulated CD8 +ve CTLs (Alexander-Miller *et al*, 1998). As CD20 is present at approximately 50 000 copies per B cell (Marti *et al*, 1992), saturated binding of HLA class I/peptide complexes could result in 50 000–200 000 copies of a single peptide/HLA class I combination per cell. This is significantly higher than produced by peptide pulsing which results in peptide placement in 5000 copies of an individual HLA allele (Delon *et al*, 1998) out of a total allele number of 10^5^ per B cell (Kageyama *et al*, 1995; McCune *et al*, 1975). Additionally the stability of the recombinant complexes appears to be greater than those produced by peptide pulsing which have an average half life of only 2.5–4 h (Wataya *et al*, 2001) which may further increase the strength and duration of T cell activation.

The B9E9 scFvSA fusion protein is currently in clinical trials for the treatment of B cell lymphoma using radiolabelled biotin as the effector function. The ease of administration, lack of toxicity and option for repeated doses suggest that using this molecule in a vaccine strategy should be feasible. To date recombinant HLA class I molecules are yet to be administered to cancer patients, however as endogenous HLA class I molecules circulate in health and in increased levels in a number of illnesses (McMillan *et al*, 1997) they are unlikely to have any major direct toxicity.

In this preliminary work we have focused on stimulation with a single HLA class I/peptide complex, however the ability to make these recombinant molecules of any chosen HLA class I/peptide combination should allow for vaccination with a range of complexes either sequentially or concurrently. As the stability of the HLA class I/peptide complexes appears to vary considerably with the identity of the peptide (Valmori *et al*, 1998) and stability is closely linked to the immunogenicity of a chosen HLA class I/peptide complex, it is possible that recombinant molecules that incorporate the peptide/HLA heavy chain/beta-2-microglobulin into fusion proteins may offer potential benefits.

The initial clinical studies will use PBMCs targeted with complexes *ex vivo*, which will allow accurate administration of designated numbers of targeted cells at the optimum epitope density. This approach should also minimise the potential immunogenicity of the streptavidin in the fusion protein and the potential risk of uncontrolled CTL expansion that could occur with intravenous HLA classI/complex administration that would result in the targeting of the total B cell population.

The ability of antibody targeted HLA class I/peptide complexes to specifically induce the expansion of CTLs to a single specificity should prove useful for *in vitro* studies analysing the endogenous CTL response or the effects of other *in vivo* procedures. Potentially this system could also be used for the *ex vivo* production of CTLs for the adoptive immunotherapy of cancer and other diseases. However a vaccination procedure based on targeting HLA class I/peptide complexes to B cells *in vivo* via the antibody delivery system could offer significant advances in both the applicability and effectiveness of cancer vaccines.
